# Synergistic Effects of Nanochitosan and Demineralized Dentin Matrix on Alveolar Bone Substitution

**DOI:** 10.1590/0103-644020256759

**Published:** 2026-01-12

**Authors:** Renie Kumala Dewi, Sri Oktawati, Asdar Gani, Eko Suhartono, Nurlindah Hamrun, Rasmidar Samad, Nurhayaty Natsir, Maharani Laillyza Apriasari, H. Hasanuddin

**Affiliations:** 1 Doctoral Program, Faculty of Dentistry, Hasanuddin University, 90245, Makassar, Indonesia; 2 Department of Pediatric Dentistry, Faculty of Dentistry, Lambung Mangkurat University, 70122, Banjarmasin, Indonesia; 3 Department of Periodontology, Faculty of Dentistry, Hasanuddin University, 90245, Makassar, Indonesia; 4 Department of Medical Biochemistry, Faculty of Medicine, Lambung Mangkurat University, Indonesia; 5 Department of Oral Biology, Faculty of Dentistry, Hasanuddin University, 90245, Makassar, Indonesia; 6 Department of Public Dental Health, Faculty of Dentistry, Hasanuddin University, 90245, Makassar, Indonesia; 7 Department of Conservative Dentistry, Faculty of Dentistry, Hasanuddin University, 90245, Makassar, Indonesia; 8 Department of Oral Medicine, Faculty of Dentistry, Lambung Mangkurat University, 70122, Banjarmasin, Indonesia; 9 Master of Dental Sciences Study Program, Faculty of Dentistry, Hasanuddin University, 90245, Makassar, Indonesia

**Keywords:** Bone Remodeling, Black Soldier Fly Pupae, Demineralized Dentin Matrix, Nanochitosan, Tooth Graft

## Abstract

The purpose of this study was to evaluate the effectiveness of a combination of nanochitosan, Black Soldier Fly (BSF) (*Hermetia illucens)* pupae, and Demineralized Dentin Matrix (DDM) from human teeth in forming Receptor Activator of Nuclear Factor Kappa-B Ligand (RANKL), Osteoprotegerin (OPG), osteoblasts, and osteoclasts after tooth extraction in guinea pigs. A sample of 18 guinea pigs (*Cavia cobaya*) was randomly assigned to two groups: the treatment group (T) (n=9) and the control group (C) (n=9). The left mandibular incisor of the guinea pigs was extracted, after which the socket in the control (C) group was applied with polyethylene glycol gel as a homogeneity treatment, followed by suturing with non-absorbable silk, In the treatment (T) group, a gel composed of nanochitosan BSF pupae and DDM (NBSF+DDM) was applied to the socket, followed by suturing with non-absorbable silk. The samples were euthanized, and the mandibles were dissected on days 7, 14, and 21 to observe the number of osteoblasts and osteoclasts, and then the expression of OPG and RANKL, which are key biomarkers of bone regeneration. The results of this study showed an increase in the number of osteoblasts and the expression of OPG, a decrease in the number of osteoclasts and the expression of RANKL. The combination of nanochitosan BSF pupae and DDM from human teeth affects increasing the expression of OPG and osteoblasts, while decreasing RANKL and osteoclasts on the 7th, 14th, and 21st days, and may help improve bone remodeling.

## Resumo

O objetivo deste estudo foi avaliar a eficácia da combinação de nanoquitosana, pupas de mosca soldado-negra (Black Soldier Fly - BSF) (Hermetia illucens) e Matriz de Dentina Desmineralizada (DDM) proveniente de dentes humanos na formação do Ligante do Receptor Ativador do Fator Nuclear Kappa-B (RANKL), Osteoprotegerina (OPG), osteoblastos e osteoclastos após a extração dentária em cobaias. Uma amostra de 18 cobaias (Cavia cobaya) foi distribuída aleatoriamente em dois grupos: o grupo tratamento (T) (n=9) e o grupo controle (C) (n=9). O incisivo mandibular esquerdo das cobaias foi extraído; em seguida, o alvéolo do grupo controle (C) recebeu gel de polietilenoglicol como tratamento de homogeneização, seguido de sutura com fio de seda não absorvível. No grupo tratamento (T), um gel composto por nanoquitosana, pupas de BSF e DDM (NBSF+DDM) foi aplicado ao alvéolo, seguido de sutura com fio de seda não absorvível.As amostras foram sacrificadas e as mandíbulas dissecadas nos dias 7, 14 e 21 para observar o número de osteoblastos e osteoclastos, além da expressão de OPG e RANKL, que são biomarcadores essenciais da regeneração óssea. Os resultados deste estudo mostraram um aumento no número de osteoblastos e na expressão de OPG, além de uma redução no número de osteoclastos e na expressão de RANKL. A combinação de nanoquitosana, pupas de BSF e DDM de dentes humanos influencia o aumento da expressão de OPG e de osteoblastos, ao mesmo tempo em que reduz RANKL e osteoclastos nos dias 7, 14 e 21, podendo contribuir para a melhora do remodelamento ósseo.

**Figure:**
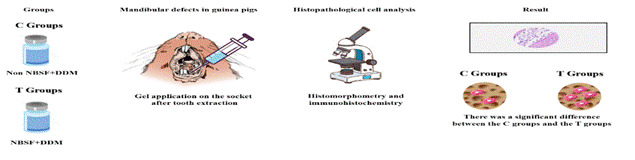

## Introduction

Several studies have reported that alveolar bone loss due to resorption following tooth extraction is estimated at 31.6% after 3 months, 42.4% after 6 months, and 50.73% after 12 months. Bone remodeling is a phase in which damaged bone is resorbed by osteoclasts, followed by the deposition of new bone matrix by osteoblasts to maintain skeletal integrity and function. Excessive bone loss impairs the bone's self-repair capacity, thereby necessitating the use of supportive materials to promote healing, prevent infection, and facilitate bone regeneration, such as bone grafts^1^ ^,^ ^2^.

Bone grafts can enhance the bone healing response by supplying osteogenic cells and promoting osteoinductive and osteoconductive activity. Bone grafts vary based on their source and are classified into three categories: autografts, allografts, and xenografts. Autografts are considered the gold standard due to their excellent osteogenic and osteoconductive properties. However, they present certain limitations, such as limited availability, donor site morbidity, and unpredictable resorption rates. Consequently, various allogeneic substitute materials have been developed as alternative candidates to stimulate osteoblast migration and proliferation^3^ ^,^ ^4^.

In bone tissue engineering innovations, three-dimensional scaffolds have been developed that can be absorbed by the body, similar to polymers, which can accelerate the replacement of damaged tissues or function as extracellular matrices. A polymer that is frequently employed is chitosan. Beetles and silkworms are among the insects commonly used as sources of chitin. From the prepupal stage to the pupae stage, the exoskeleton has 35% chitin content. Other potential sources of insect chitin can also be found in Black Soldier Fly (BSF) (*Hermetia illucens)* pupae and shells, where chitin can be processed into chitosan. Chitin can be converted to chitosan through the process of deacetylation. The characteristics of chitosan obtained are highly dependent on the effectiveness of the deacetylation stage and the source of chitin used^5^ ^,^ ^6^.

Based on the experimental results of secondary metabolite compound testing on BSF pupae, the following findings were obtained: the hexane extract solution contained alkaloids, flavonoids, saponins, terpenoids, and triterpenoids. The ethyl acetate and methanol extract solutions were found to contain alkaloids, saponins, and terpenoids. The IC₅₀ values obtained for the n-hexane, ethyl acetate, and methanol extracts were 41.18 mg/L, 36.92 mg/L, and 17.45 mg/L, respectively, indicating that the methanol extract exhibited the highest bioactivity among the tested solvents and indicating that BSF pups possess potent antioxidant qualities. Natural antioxidants found in animals are glutathione (GSH, γ-glutamyl-cysteinyl-glycine) and various enzymes that can eliminate ROS (reactive oxygen species) excess, such as catalase, and enzymes that use GSH as a substrate (GSH-reductase and GSH-peroxidase)^7^ ^,^ ^8^.

Nanoparticles have advantages over similar larger materials because their size provides a larger surface area-to-volume ratio, making them more reactive. Nanochitosan, which is a small part of chitosan, is formed from repeating units of 2-amino-2-deoxy-D-glucose linked by β- ^1^ ^,^ ^2^ ^,^ ^3^ ^,^ ^4^-glycosidic bonds, forming a monomer chain similar to glycosaminoglycan (GAG). GAG is also known as a mucopolysaccharide that plays a vital role in wound healing^9^. Based on the study by Mahmoud et al. (2021), it was concluded that rats treated with nanochitosan exhibited successful osteoblast migration in diseased bone and showed a higher OPG/RANKL ratio as well as increased BMP-2 expression levels compared to those not receiving nanochitosan. Furthermore, osteoblast implantation was found to inhibit bone resorption and improve bone histomorphology^10^ ^,^ ^11^.

However, chitosan has weaknesses, such as low mechanical strength and a lack of active sites that can enhance the membrane's working function. To overcome these weaknesses, it is necessary to modify one of them with other natural materials, such as the DDM. DDM can be derived from human teeth dentin, possessing a composition nearly comparable to that of bone, including 2% non-collagenous protein, 10% body fluid, 18% collagen, and 70% hydroxyapatite. Dentin can trigger osteoinduction, osteoconduction, and blood vessel formation due to the presence of several growth factors. ^12^ ^,^ ^13^. The advantages of using DDM derived from human dental dentin include osteoconductive and osteoinductive properties, reduced anesthetic use, increased surgical time efficiency, reduced blood loss, and the absence of surgery on other parts of the patient's body. ^14^. According to the study by Grawish et al. (2022), DDM has demonstrated potential in accelerating osteoblast differentiation and promoting OPG expression in animal studies ^15^.

One of the novelties of this study lies in the use of nanochitosan, which has been widely investigated for its role in bone remodeling, as indicated by various biomarkers of bone formation. However, this study uniquely explores the combination of nanochitosan-derived BSF pupae with DDM to accelerate the formation of alveolar bone substitutes. The evaluation of this combination in promoting bone regeneration biomarkers following tooth extraction has not been previously reported. This study aimed to evaluate the effectiveness of a combination of nanochitosan, BSF pupae, and DDM from human teeth in inducing the formation of RANKL, OPG, osteoblasts, and osteoclasts after tooth extraction in guinea pigs.

## Materials and methods

### Protocol study

This research was conducted by following the guidelines of the Ethics Committee for Teaching and Research on Animals at RSGMP Hasanuddin University with number 0108/PL.09/KEPK FKG-RSGM UNHAS/2023.

### Preparation of gel materials

This research utilizes BSF pupae obtained from CV Maggo Banua, South Kalimantan, Indonesia, to process into chitosan and nanochitosan. The initial phase involved the production of chitosan BSF pupae, which included the processes of demineralization, deproteinization, depigmentation, and deacetylation. The demineralization process involved soaking dried and powdered BSF pupae in 3M hydrochloric acid at a 1:10 (w/v) ratio at room temperature. The samples were then thoroughly rinsed with sterile distilled water until a neutral pH was reached, followed by oven drying at 60°C. Deproteinization was subsequently performed by treating the resulting residue with 2M sodium hydroxide at a 1:10 (w/v) ratio at room temperature. Depigmentation was performed by immersing the deproteinized residue in a 2% potassium permanganate (KMnO₄) solution at a 1:10 (w/v) ratio, followed by immersion in 2% oxalic acid at the same ratio. This process yielded chitin from the BSF pupae, which was then deacetylated to convert the chitin into chitosan. The deacetylation of chitin was conducted by immersing it in a 50% NaOH solution (1:10) at 80°C for 12 hours with the aid of a magnetic stirrer. Deacetylation is a reaction that releases acetyl groups (-COCH3) in chitin using a base solution to form free amino groups (-NH2), which are characteristic of the chitosan structure. The chitosan derived from BSF pupae has a deacetylation rate of 80%, which indicates high purity in chitosan BSF pupae. To prepare nanochitosan, the chitosan powder was dissolved in 50 mL of 1% acetic acid, transferred to a beaker, and stirred using a magnetic stirrer to achieve complete dissolution. A 0.1% sodium tripolyphosphate (NaTPP) solution was then added at a chitosan-to-TPP ratio of 5:1, resulting in the formation of a powder with an average size of 204.9 nm. Demineralized Dentin Matrix (DDM) is prepared from extracted human teeth, produced by selecting the roots of the teeth, and then grinding through a planetary ball mill PM 100-Japan to produce particles measuring 500-1000 microns. The process continues with demineralization; the dentin powder is immersed in 1% hydrochloric acid (HCl) for 2 hours, followed by thorough rinsing with distilled water and drying. The next step is to freeze-dry (Isomag-USA) the sample for 18-24 hours, followed by sterilization using gamma rays.

Gel preparation that will be applied to the post-tooth extraction socket of guinea pigs by mixing nanochitosan BSF pupae powder (NBSF) + Demineralized Dentin Matrix (DDM) powder (ratio 50:50) and PEG 400 and PEG 4000 until homogeneous. The sample was divided into two groups: the control group (K), which received no combination of NBSF+DDM gel, and the treatment group (K), which received a combination of NBSF+DDM gel.

### Sample size

This study used guinea pig samples obtained from the Faculty of Veterinary Medicine, Airlangga University. A total of eight juvenile male guinea pigs (Cavia cobaya) were included. The inclusion criteria included healthy, active animals aged 3-4 months, and weighing between 300 and 375 grams. The exclusion criteria were animals that died before the study. All animals were confirmed to be healthy through physical examination by a veterinarian. The animals to be studied were adapted for seven days, during which guinea pigs were provided with food and water by standardized protocols, which continued throughout the experiment's duration.

### Sample treatment procedures

Guinea pigs were anesthetized intramuscularly using a dose of ketamine (50 mg/kg body weight) and a dose of xylazine (5 mg/kg body weight) (2% Alfazyne, Alfasan International, Woerden, Netherlands). Tooth extraction was performed on the left mandibular incisor, and the socket was irrigated with sterile distilled water to remove any remaining debris left in the tooth extraction socket. In the control group (C) (n = 9), after tooth extraction, the wound was sutured with non-resorbable silk. The treatment group (T) received NBSF+DDM gel after tooth extraction, which was applied into the post-extraction socket using a sterile plastic syringe until it was filled (±0.2 ml). The wound was then sutured with non-resorbable silk. Euthanasia was performed on days 7, 14, and 21 post-treatment. The euthanasia process begins with an intraperitoneal injection of a lethal dose of ketamine (4 times the anesthetic dose or 0.4 mL/400 g body weight) into the lower abdomen until the guinea pig dies. The tissue of the euthanized guinea pig is removed by vertically cutting the mandible in the post-extraction socket region in the mesial and distal regions. The left mandible of each guinea pig was then subjected to a five μm thick sagittal section using a microtome (Sakura Finetek, USA) at the tooth socket site after extraction of the lower left incisor, using a dental bur to prepare the tissue specimen. After confirming the death of the animal, the guinea pig was subsequently readied for interment.

### Histochemical analysis

Tissue specimens were taken from the mandibles of guinea pigs using a scalpel and cut in the interdental area of the mandibular incisors. After the tissue was obtained, the specimens were stored in a formalin solution to prevent tissue changes, including decay, hardening, and alterations in the refractive index of various tissue components, as well as increased tissue affinity for the dye. After the first 48 hours, the fixative solution was able to penetrate evenly into the tissue. In the second stage, the specimens were left in the solution for 48 hours. After fixation, the tissue was rinsed with running water for 6-9 hours. Then, decalcification was performed, a process of removing calcium with a solution that binds calcium ions, namely, using a 10% EDTA solution at a neutral pH for approximately 1.5 months until the mandibular tissue was soft. Every 1 week, the 10% EDTA solution was replaced with a new one, placed in a 5% HNO3 decalcification solution for 1 hour. Histological tissue section staining was performed using Hematoxylin and Eosin (HE) stain (HE09-40R, TissuePro, USA). The expression levels of OPG and RANKL were evaluated using immunohistochemistry (IHC), using a monoclonal antibody specific for RANKL and an OPG-specific monoclonal antibody (sc-7628, Santa Cruz Biotechnology) on bone tissue sections. Histological observation of the area of the apical third of the post-extraction socket to see the number of osteoblasts and osteoclasts, as well as the expression of OPG and RANKL, using a light microscope at the Research Center Laboratory, Faculty of Dental Medicine, Universitas Airlangga. Microscopic observations were performed using an Olympus CX23 (China) photomicroscope, equipped with OlyVIA (Viewer for Imaging Applications) software, at 400x magnification for each field of view. The researcher, a laboratory analyst and a specialist in oral pathology, conducted the analysis. The measurements were validated through analysis using Image software.

### Statistical analysis

Data were obtained by examining histological sections stained with HE and IHC using a light microscope at 400x magnification, and manually counting the number of osteoblasts, osteoclasts, as well as the expression of OPG and RANKL in the alveolar bone. This study did not perform radiographic analysis or computed microtomography due to the long waiting time for reading the photos and not using a digital system (teleradiology). However, this will be done in future studies. Data were compiled using Microsoft Excel, and statistical analyses were performed using SPSS software version 25.0 (IBM Corp., Chicago, IL, USA). The data obtained were analyzed using One-Way ANOVA (p < 0.05).

## Results

### Quantitative analysis based on data collection results

**Figure 1:**
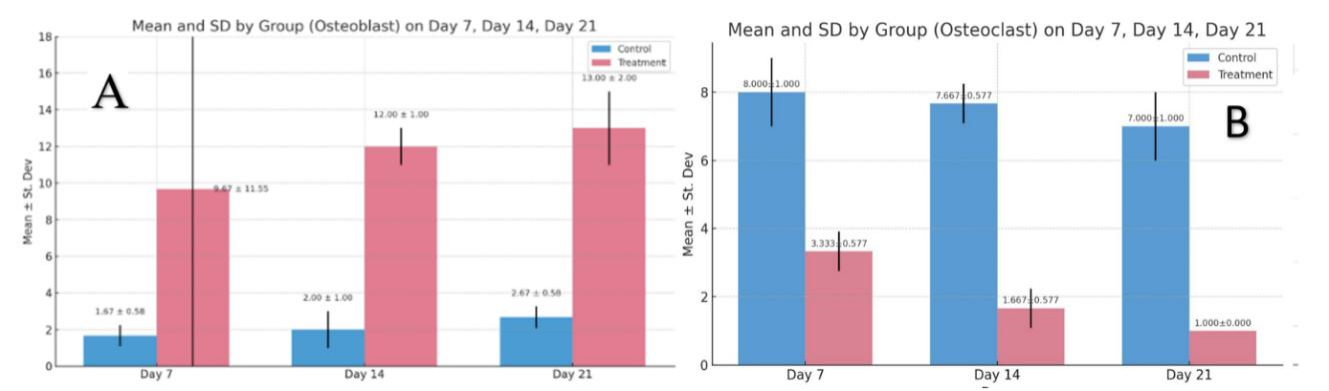
Graph of measurement results: (A) number of osteoblasts; (B) number of osteoclasts, on days 7, 14, and 21 in the C group and T group.

**Figure 2:**
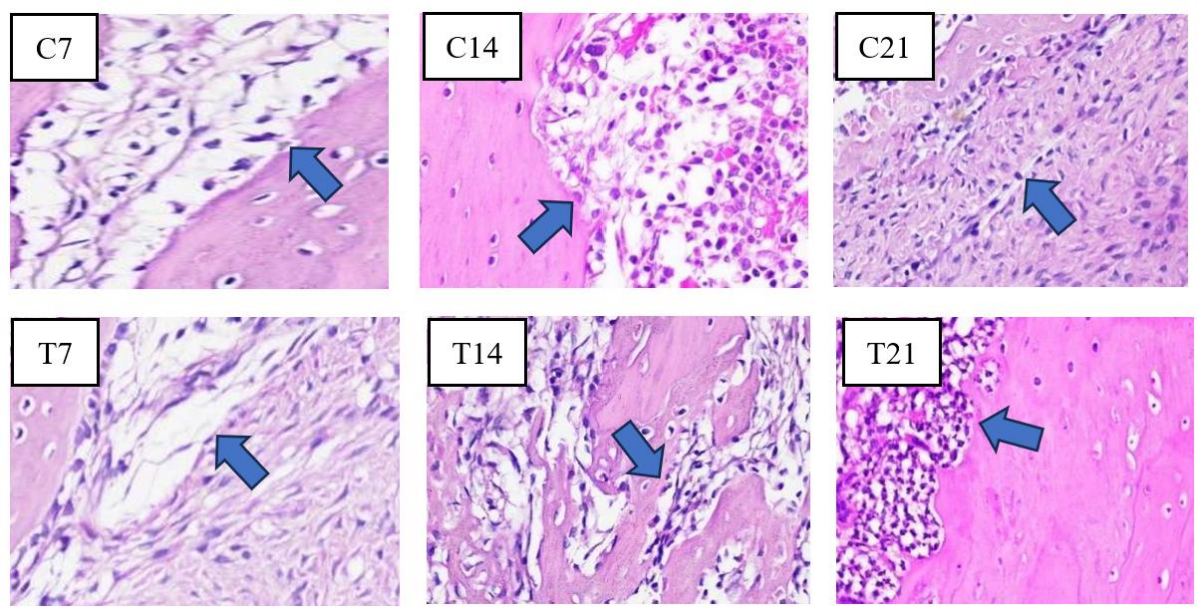
Overview of HE of osteoblasts showing cuboidal to cylindrical in shape with a diameter of 20-30 μm and a purplish color observed in the apical third of the edge of the socket of guinea pig (Cavia cobaya) teeth post-extraction in the T group and C group under a light microscope at 400x magnification on days 7, 14, and 21.

The results of the study on samples from the control group (C) and the treatment group (T) after tooth extraction on days 7, 14 and 21 respectively showed that the T group had the highest increase in the number of osteoblasts on day 21 (13,000 ± 2,0000) and the highest OPG expression on day 21 (11,333 ± 1,000) compared to the C group. In the T group, there was also the most significant decrease in the number of osteoclasts on day 21 (1,000 ± 0.0000) and RANKL expression on day 21 (1,000 ± 1.0000) (Figures 1 and 2).

### Histopathological cell analysis in the alveolar bone of guinea pigs

**Figure 3:**
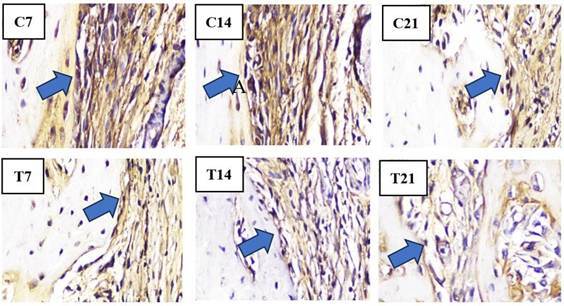
Overview of IHC of OPG showing the amount of OPG expressed by osteoblasts in bone preparations by providing a positive brownish reaction in the apical third of the edge of the socket of guinea pig (*Cavia cobaya*) teeth post-extraction in the T group and C group under a light microscope at 400x magnification on days 7, 14, and 21.

In the histopathological anatomical results, cell counting was performed using a light microscope to identify osteoblasts, osteoclasts, OPG, and RANKL. Light micrographs of sections stained with HE and IHC revealed a higher presence of osteoblasts (Figure 2) and OPG expression (Figure 3) along the socket margin in the T group compared to the C group. From a microscopic perspective, osteoblasts are cuboidal to cylindrical cells that have a purplish coloration and a diameter of 20-30 μm and exhibit a purplish coloration located on the surface of the bone matrix, and are arranged adjacent to one another. and arranged adjacent to one. In OPG expression, a positive brownish reaction occurs as a result of IHC examination with anti-OPG monoclonal antibodies.

**Figure 4:**
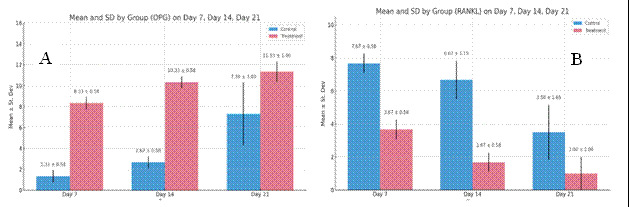
Graph of measurement results: (A) OPG expression; (B) RANKL expression on days 7, 14, and 21 in the C group and T group.

The results of the study on samples from the control group (C) and the treatment group (T) after tooth extraction on days 7, 14, and 21 respectively showed that the T group had the highest increase in the number of OPG on day 21 (11,33 ± 1,00) compared to the C group. In the T group, there was also the most significant decrease in RANKL expression on day 21 (1,000 ± 1,000) compared to the C group (Figure 4).

**Figure 5:**
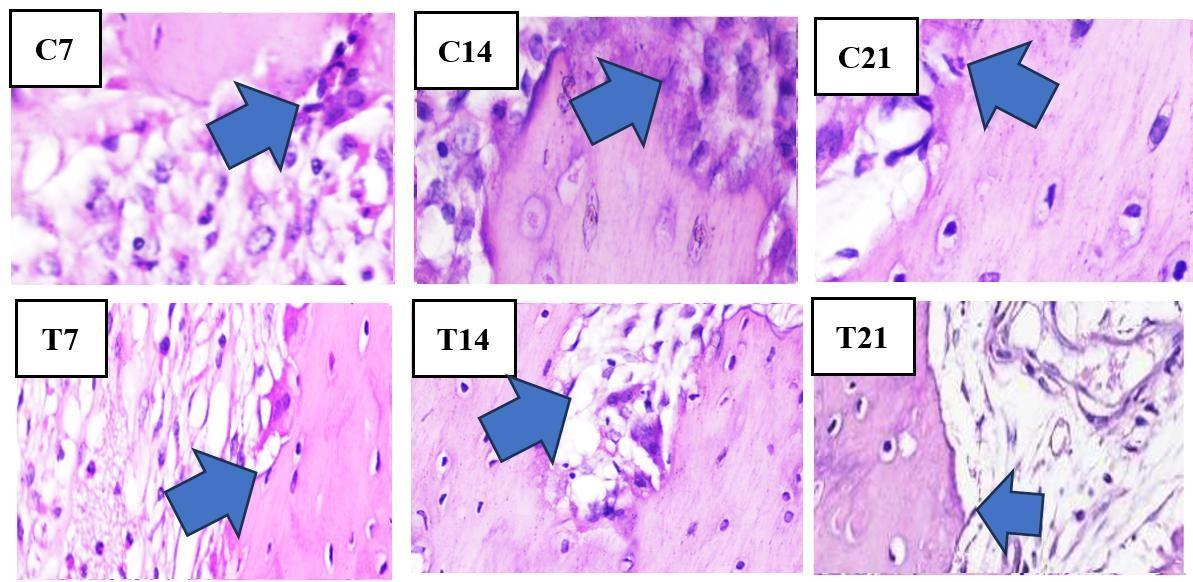
Overview of HE of osteoclast showing large, motile cells with many nuclei in the apical third of the edge of the socket of guinea pig (*Cavia cobaya*) teeth post-extraction in the T group and C group under a light microscope at 400x magnification on days 7, 14, and 21.

**Figure 6:**
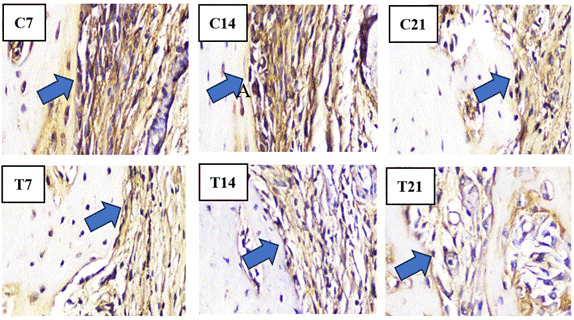
Overview of IHC of OPG showing the amount of OPG expressed by osteoblasts in bone preparations by providing a positive brownish reaction in the apical third of the edge of the socket of guinea pig (*Cavia cobaya*) teeth post-extraction in the T group and C group under a light microscope at 400x magnification on days 7, 14, and 21.

Conversely, the C group exhibits the highest concentrations of osteoclasts and RANKL as opposed to the T group around the socket (Figures 5 and 6). Osteoclast cells are large, multinucleated cells that play a crucial role in matrix resorption during bone growth and remodeling. RANKL expression is indicated by a positive brownish staining, as detected through immunohistochemical analysis using monoclonal anti-RANKL antibodies.

Based on data analysis, significant differences were found between the control group (K) and the treatment group (T) (0.000) (p<0.005) in osteoblasts, osteoclasts, OPG, and RANKL using the one-way ANOVA test.

**Figure 7:**
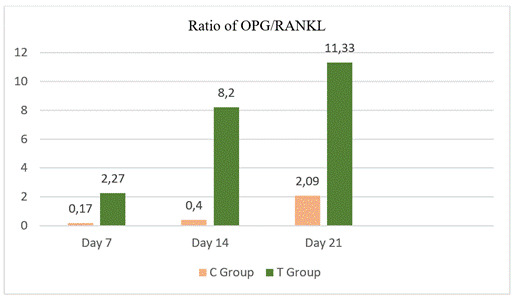
Ratio of OPG/RANKL in the C group and T group on days 7, 14, and 21.

This mechanism suppresses osteoclastogenesis and inhibits the development of osteoclasts. Based on Figure 7, the OPG/RANKL ratio was higher in group T than in group C. The treatment group with NBSF+DDM on day 21 had the highest OPG/RANKL ratio at 11.33, while the control group had the lowest OPG/RANKL ratio on day 7 at 0.17. This means that the NBSF+DDM application group on day 21 had the highest OPG expression.

## Discussion

The results of the combination of Nanochitosan BSF pupae and DDM showed an increase in osteoblast numbers and OPG expression, a decrease in osteoclast numbers and RANKL expression, when compared to both the control (C) group and the treatment (T) group on days 7, 14, and 21. This study showed a significant difference between the control (C) group and the treatment (T) group, attributed to the ability of chitosan to suppress the production of proinflammatory cytokines, including IL-1, IL-6, and TNF-α. These cytokines are involved in osteoclast formation and activation through the RANK/RANKL signalling pathway. Upon interaction with inflammatory cells, chitosan releases N-acetyl-D-glucosamine (NAG), which subsequently binds to specific receptors on macrophages, contributing to its anti-inflammatory effects. This process stimulates the recruitment of inflammatory cells to the wound site and promotes their proliferation within the affected area. The increased presence of inflammatory cells facilitates the release of anti-inflammatory cytokines and growth factors, thereby supporting cellular proliferation and tissue regeneration. Some of the cytokines and growth factors that play an important role in the wound epithelialization process are from the Epidermal Growth Factor (EGF) and Heparin-Binding EGF (HB-EGF); FGF family, Transforming Growth Factor β1 (TGFβ1); and KGF (Keratinocyte Growth Factor) ^16^.

The findings of this study are consistent with in vitro research by Kazimierczak (2021), pada hari ke 7 dan 21, which demonstrated that macrophages cultured on chitosan substrates for seven days exhibited significantly elevated levels of TGF-β1 and IL-4 compared to M0, M1, and M2 macrophage phenotypes. High levels of TGF-β1 increase the expression and secretion of osteoprotegerin (a decoy receptor for the receptor activator ligand of nuclear factor kappa-Β (RANKL)), which suppresses RANKL-RANK-mediated osteoclastogenesis. This study also showed a decrease in RANKL expression on days 7, 14, and 21 after application of BSF pupae nanochitosan. Nanochitosan BSF pupae can activate key inflammatory cells, including macrophages, polymorphonuclear leukocytes (PMNs), and fibroblasts, thus playing a critical role in modulating inflammation and supporting tissue regeneration. Chitosan has been shown to directly activate multipotent mesenchymal progenitor cells and osteogenic cells, thereby promoting their differentiation into osteogenic lineages. This property underscores its potential in accelerating bone regeneration. In addition, the flavonoid content in BSF pupae can inhibit prostaglandin synthesis, PGE-2, which reduces macrophage infiltration, plays a role in stimulating osteoclast formation directly or indirectly through RANKL, resulting in differentiation and fusion of osteoclast precursors into osteoclasts. Therefore, the presence of PGE-2 inhibition and cytokine synthesis can function as an inhibitor of osteoclast formation, so that the number of osteoclast cells and proinflammatory cytokines can also inhibit osteoprotegerin (OPG). In other words, decreased PGE-2 synthesis indirectly induces bone formation only by activating the biological cascade of osteoblastogenesis by inactivating RANKL in wound healing after extraction ^17^ ^,^ ^18^.

In this study, nanochitosan-based BSF pupae were reported to have a high deacetylation rate of around 80%. Hemostasis begins to occur due to a higher degree of deacetylation (DD) of chitosan, thereby increasing the aggregation of erythrocytes and platelets, which are essential for the hemostasis process. The chitosan used in this study exhibited a high degree of deacetylation (80%). The chitosan used in this study exhibited a high degree of deacetylation (80%). Consistent with Hakim's (2022) findings, chitosan with a higher degree of deacetylation exhibited superior antibacterial efficacy compared to chitosan with a lower degree of deacetylation. A higher degree of deacetylation correlates with greater availability of active chitosan molecules that inhibit bacterial growth.

According to Dewi (2024), chitosan from BSF pupae with a DD value of 80% indicates good quality. The higher the DD, the higher the quality of chitosan. The duration and temperature of deacetylation influence the DD value; higher temperatures increase intermolecular motion, thus accelerating the rate of the acetyl group cleavage reaction. ^19^ ^,^ ^20^ ^,^ ^21^.

The results of the study showed that the T group, treated with the NBSF+DDM combination, had the highest average OPG expression compared to the C group on the -7th, -14th, and -21st days, because NBSF+DDM contained DDM. DDM derived from human teeth has the same biochemical composition as bone, which is composed of both organic and inorganic elements. The composition of bone and dentin is almost the same, including 2% non-collagenous protein, 10% body fluid, 18% collagen, and 70% hydroxyapatite. Bone and dentin also contain several growth factors and proteins, such as FGF (fibroblast growth factor), collagen types I and III, and BMP (bone morphogenetic protein), which are also found as matrix proteins included in non-collagenous proteins. Several growth factors in dentin can trigger osteoinduction, osteoconduction, and vascular formation ^21^ ^,^ ^22^. This study aligns with Ding's (2020) conclusion that DDM administration can enhance new bone formation and increase the ratio of alveolar bone height and density in the area where the dental implant is placed, as observed in the fourth week ^23^ ^,^ ^24^. The acceleration of bone remodelling is derived from the biological effects of OPG on bone cells, including inhibition of the final stage of osteoclast differentiation, suppression of mature osteoclast activation, and induction of apoptosis. Therefore, the OPG/RANKL balance can control the acceleration of bone remodelling. Osteoblasts produce OPG, which acts as a RANKL receptor, preventing RANK from binding to RANKL and inhibiting osteoclastogenesis. Consequently, when RANKL binds to OPG, osteoclast development does not occur. The OPG/RANKL ratio is used as an indicator of bone formation or resorption. The RANKL/RANK/OPG system is well-recognised for its role in osteoclast maturation, bone modelling, and bone remodelling. OPG and RANK are receptors that exhibit an equal affinity for RANKL. OPG, produced by osteoblasts, functions as a decoy receptor for RANKL, preventing its binding to RANK and subsequently inhibiting RANK activation ^23^ ^,^ ^24^ ^,^ ^25^ ^,^ ^26^.

Considering the limitations of this study, it is necessary to investigate the acceleration of bone remodeling by examining other bone formation markers besides OPG, RANKL, Osteoblasts, and osteoclasts, such as BMP-2, osteocalcin, alkaline phosphatase, and evaluating the observation time for more than 21 days.

## Conclusion

Bone remodeling research using a combination of BSF nanochitosan and DDM pupae has shown an increase in osteoblast number and Osteoprotegerin (OPG) expression, a decrease in osteoclast number and RANKL on days 7, 14, and 21, both biomarkers of bone formation.

The BSF nanochitosan pupae contain flavonoids and 2-amino-2-deoxy-D-glucose linked by β-^1^ ^,^ ^2^ ^,^ ^3^ ^,^ ^4^-glycosidic bonds, forming a monomer chain similar to glycosaminoglycans that can accelerate wound healing. Meanwhile, DDM, a human tooth compound, contains growth factors, making this material synergistic and able to accelerate the alveolar bone remodeling process after tooth extraction.
